# Animalculæ in the Atmosphere of Cholera Patients

**Published:** 1850-01

**Authors:** R. D. Mussey

**Affiliations:** Professor of Surgery in the Medical College of Ohio, and first Surgeon of the Commercial Hospital at Cincinnati


### Part 3.—Selections.

### ARTICLE I.

*Animalculæ in the Atmosphere of Cholera Patients.*—By R. D. Mussey, M.D., Professor of Surgery in the Medical College of Ohio, and first Surgeon of the Commercial Hospital at Cincinnati.

Several years ago, Count Moscati, of Milan, was commissioned by the government to analyse the atmosphere of the Milanese rice-grounds, and to ascertain the properties of the exhalations which arose from them. He found nothing peculiar during the day, but by suspending, through the night, glass globes filled with ice, at the height of three feet above the surface of the soil of a rice-field, he collected a fluid condensed upon the globes; which, on being kept a few days in phials, presented a flaky matter upon its surface. This appeared like mucus, and exhaled a fœtid odor. The same experiment was made in the hospital at Milan, and a similar mucosity was condensed upon the refrigerated vessels. Mr. R. Herman, of Moscow, in the first visitation of cholera in Russia, "found the air surrounding the patients to contain *a substance* which, when deposited upon cool substances, *resembled animal mucus*. It did not react upon test-papers, and was precipitated by sugar of lead and tincture of galls, bearing a great analogy to the substance which Moscati separated from infected air." Dr. Adam Neale very naturally suggests, that this substance should be submitted to inspection through the microscope.

During the prevalence of cholera this season, in Cincinnati, I was too much occupied to make the investigation I had intended, should an opportunity offer, until near the close of the epidemic.

In the last days of August, I was enabled to make the following experiment: A six quart glass jar, filled with ice and set in a clean earthen basin, was placed near to the head of the bed of a cholera patient who had recently been brought into the Commercial Hospital in collapse. He died in seven hours. The jar and basin were then transferred to the bedside of another cholera patient, and allowed to remain there eleven or twelve hours. Fluid to the amount of about three ounces was obtained by the condensation of vapor, a part of which, no doubt, must have emanated from the lungs of the patients. On the first of September, in a drop of this fluid, placed under the microscope, a multitude of animalcules were discovered moving in all directions. They were different in shape and size. The largest were somewhat oval shaped, blunt pointed at each end, the long and short diameters being nearly as three to two. The smaller ones were circular or globular; and many times less than the oval ones. The several specimens of the oval variety differ greatly as to size. I observed two or three very large ; they looked plump and moved more sluggishly than the rest, as if they might be about to multiply. The long diameter of the largest appeared to be just about equal to four diameters of a corpuscle of human blood. The motions of both varieties were performed with great apparent facility, and some of them moved with surprising swiftness across the field of the microscope.

Since the foregoing observations were made, we have obtained, at different times, three additional specimens of fluid from vapor condensed by the side of three cholera patients. In one of these specimens, animalcules were observed. The patient had a mild form of the disease, and was rapidly convalescent at the time the experiment was made. The thin, pale discharges from the bowels, however, in this case, contained animalcules. In the other two specimens of condensed fluid, a plenty of these animals were found, as well as in the rice-water discharges. Both the oval and globular animalcules were observed in each of the specimens of condensed fluid, with the one exception just mentioned. In the rice-water discharges, the globular animals seemed to predominate, but in two specimens the oval variety was pretty abundant. In the last specimen, obtained four days ago, a long, slender animal is also found in the rice-water discharges. Viewed through a magnifier of two thousand linear diameters, he appears to be about *one-fourth* of an inch long—his real length being $\frac{1}{8000}$ of an inch. Is it a vibrio? He moves deliberately with a lateral flexure of his body, like a serpent on the ground, not with the up and down motion alleged to belong to the supposed sea-serpent of the Atlantic ocean. My son, Dr.

Wm. H. Mussey, who has greatly aided in these investigations, is confident that he has found this serpent-shaped animalcule in our last specimen of fluid condensed from the atmosphere. . He has seen it several times, on succesive trials of the fluid. Many of the specimens of the globular rice-water animalcules appeared to be not more than *one-twentieth* of the diameter of a blood corpuscle, while some of the oval variety, as already suggested, were in their long diameter, equal to four blood corpuscles, making a difference of eighty diameters in the size of the two kinds. In the oval variety of the first specimen obtained, I observed one of the animalcules to make a number of rapid gyrations in a small circle, then to poise himself upon one end, with his long diameter exactly vertical, then to whirl himself for some time with great velocity upon this vertical axis like a spin-top What was the object of this remarkable flourish, I could not satisfy myself. It was the only instance of the kind which was witnessed.

These animals exhibit a considerable degree of tenacity of life. When collected upon the surface of the refrigerated vessel, they were compelled to endure the temperature of the freezing point of water ; and they were afterwards very active at nearly eighty degrees Fahrenheit. Probably their range of temperature, compatible with life, is much greater than this. The atmospheric animalcules of the specimen obtained the last days of August, survived thirteen days in a phial loosely corked, and the rice-water animals of the second specimen procured early in September, were still alive in considerable numbers, fourteen days after.

A shred of the vastus externus muscle, taken from a cholera patient ten hours after death, and inspected through the microscope, after having been for a few moments moistened with distilled water, exhibited mutiludes of globular animalcules. Animal as well as vegetable substances undergoing decomposition, are generally understood to swarm with animals which seem to take an important part in pulling down the organized fabric, and resolving its materials into their primitive elements; but we were not prepared to meet with them so early, even before there was the least smell of change. We tried the same experiment with a piece of muscle taken twelve hours after death, from a subject dead of erysipelas engrafted upon a broked down constitution, without being able to detect a single animalcule.

We have also failed in finding these animals in the water used by the patients at the hospital. This is the hydrant water, brought from the Ohio river in iron pipes and distributed through leaden tubes. Some days after the cholera had disappeared from the hospital, a quantity of the atmospheric vapor of the same ward was condensed by a refrigerator in the manner already described. In this fluid we could discover nothing like animalcules,

The foregoing exhibitions have not been limited exclusively to my son and myself; Professor L. M. Lawson has had opportunity of observing both the atmospheric and intestinal animals; so also has Mr. Foster, whose familiarity with the manipulations of the microscope was obligingly afforded us at the commencement of our researches. Whether the varieties of animals we have observed belong to one tribe in successive stages of development, or whether they differ permanently in form and function, we are not sufficiently acquainted with this department of science to determine.

It was a favorite opinion of Linnœus that contagious and most epidemic diseases depend for their propagation upon animalcular agency. To confirm or disprove this hypothesis will require far more investigation than the subject has, as yet, received. Some of the contagious diseases have been satisfactorily made out to occur in this way; but it would be unwarrantable to infer, from the few which are known, the many that are still unknown. The animals found to exist in the atmosphere, and in the bodies of cholera patients, may be essentially concerned in the propagation of this frightful epidemic, or they may have nothing whatever to do in this way. Should the foregoing experiments and observations, imperfect and limited as they are, lead others who have opportunity and leisure to pursue the subject until legitimate inferences, important to mankind, may be made, I shall be glad to have done even this little. If the animalcuar theory of cholera should be confirmed by a better acquaintance with the habitudes of these myriads of microscopic existences we may hope then to explain the mysterious movements of this "black death," and to learn the preventive and the remedy.

In conclusion, I may add that, within the past few days, we have discovered, in the matter discharged from a cancerous lip, animalcules of a circular form, surrounded by a wide fringed or ciliated border; and my son has found, in the smallpox pustule, animals of a different shape from any others we have observed, viz: somewhat oblong, rounded at the ends, and indented on one side like a bean. It very much resembles the kolpoda cucullus of Pritchard, but is many hundred times smaller.—*Western Lancet.*

*October* 19, 1849.

